# Aborted Sudden Cardiac Death Due to Acquired QT Prolongation: A Case Report

**DOI:** 10.7759/cureus.78496

**Published:** 2025-02-04

**Authors:** Georgios Aletras, Maria Bachlitzanaki, Eleni Eleftheriadou, Michael Pitarokoilis, Emmanouil G Foukarakis

**Affiliations:** 1 Department of Cardiology, Venizeleion General Hospital, Heraklion Crete, GRC; 2 Second Department of Internal Medicine, Venizeleion General Hospital, Heraklion Crete, GRC

**Keywords:** acquired long qt, amisulpride, ciprofloxacin tablets, drug-induced qtc prolongation, electrolytes and arrythmias, escitalopram, sudden cardiac death (scd)

## Abstract

Long QT syndrome (LQTS) is characterized by QT interval prolongation, which significantly increases the risk of malignant arrhythmias and sudden cardiac death, even in the absence of structural heart disease. While LQTS can be congenital, distinguishing it from the acquired form is crucial, though both may coexist in some cases. We present the case of a 60-year-old woman with a history of schizoaffective disorder treated with escitalopram and amisulpride and a recent prescription for ciprofloxacin for a urinary tract infection, who was admitted following a cardiac arrest. The episode was triggered by acquired QT prolongation due to the combined effects of QT-prolonging medications and hypokalemia. The patient was successfully resuscitated, and discontinuation of the offending medications (e.g., escitalopram, amisulpride, and ciprofloxacin) led to clinical stabilization with QT normalization, while other possible causes were ruled out (e.g., ischemia, thyroid disorders, etc.). This case highlights the importance of thorough medication review and early identification of individuals at risk for acquired LQTS to prevent potentially fatal arrhythmias. It also highlights the necessity of considering underlying genetic predisposition, especially in cases where QT prolongation persists despite the discontinuation of the offending agents and/or correction of other contributing factors, making genetic testing advisable in selected patients, as it will guide further management.

## Introduction

The QT interval on the electrocardiogram (ECG) reflects the period from the onset of ventricular depolarization to the completion of repolarization, representing the duration of the ventricular action potential. It is measured from the onset of the QRS complex to the tangent intersection of the T wave with the baseline. Since the QT interval varies with different heart rates, it requires correction, known as corrected QT interval (QTc). The most widely used method for QT interval correction is Bazett’s formula; however, it may be unreliable in the presence of significant R-R variability, as seen in conditions like sinus arrhythmia or atrial fibrillation. In such situations, utilizing a representative R-R interval - rather than pairing the longest QT interval with the shortest R-R interval - is crucial to avoid overestimating the QTc. Other formulas for QTc calculation include Fridericia’s, Framingham’s, and Hodges’ formulas, though they are less commonly used [1]. Clinicians should also distinguish the second component of a biphasic T-wave from a U-wave and exclude any present U-waves when measuring the QT interval [2].

A prolonged QT interval on the surface ECG is an indirect indicator of extended ventricular action potential duration (APD). This prolongation may result from an increase in inward current (e.g., sodium or calcium channels) or a decrease in outward current (e.g., potassium channels). For example, reduced potassium efflux during phase 3 of the action potential delays repolarization, while increased late sodium or calcium influx prolongs depolarization, both contributing to QT prolongation. Following puberty, a QTc of ≥450 milliseconds (ms) in men or ≥460 ms in women is widely considered borderline prolonged in most clinical guidelines, provided no significant factors interfere with accurate QTc assessment. A QTc measurement of 500 ms or longer is generally associated with an increased risk of ventricular arrhythmias, particularly torsades de pointes (TdP), a polymorphic ventricular tachycardia that can cause syncope, hemodynamic collapse, and sudden cardiac death [3-5].

Long QT syndrome (LQTS) can be classified into two main categories: congenital and acquired. Acquired LQTS is by far more prevalent than its congenital counterpart and is characterized by functional changes in ion channels without structural abnormalities. The high prevalence of acquired LQTS is largely attributed to the widespread use of QT-prolonging medications and electrolyte imbalances. This typically occurs due to the blockade of the rapid component of the delayed rectifier potassium current (IKr), the primary repolarization current of the heart. Medications known to block the IKr current include certain antiarrhythmics (e.g., amiodarone, sotalol), antibiotics (e.g., macrolides like erythromycin and fluoroquinolones like ciprofloxacin), antipsychotics (e.g., haloperidol, amisulpride), and antidepressants (e.g., escitalopram, citalopram). Blockade of the IKr current prolongs phase 3 rapid repolarization of the cardiac action potential, increasing the risk of early afterdepolarizations (EADs) by facilitating the reactivation of inward depolarizing currents, mainly via L-type calcium channels and the sodium-calcium exchange current. These EADs, arising during phase 2 or early phase 3, can trigger ventricular extrasystoles in the setting of QT prolongation observed on the surface ECG. Additionally, heterogeneity in ventricular repolarization can lead to refractoriness dispersion, creating zones of unidirectional block. Recurrent extrasystoles, combined with unidirectional block and slow conduction, may initiate reentrant circuits that culminate in TdP, which can rapidly degenerate into ventricular fibrillation (VF) - a life-threatening arrhythmia [3,5,6].

Recent reports suggest that some drugs can also increase the late sodium current, further contributing to their proarrhythmic effect [3]. Other significant causes of acquired QT prolongation include acute myocardial ischemia, electrolyte disturbances (hypokalemia, hypomagnesemia, hypocalcemia), takotsubo cardiomyopathy, autonomic failure, hypothyroidism, intracranial bleeding, pheochromocytoma, etc. [6,7].

We present a case of aborted cardiac death due to acquired LQTS, precipitated by the combined effects of three QT-prolonging drugs (ciprofloxacin, escitalopram, and amisulpride) and hypokalemia.

## Case presentation

A 60-year-old Caucasian female with a history of schizoaffective disorder managed with escitalopram 20 milligrams daily and amisulpride 800 milligrams daily, along with dyslipidemia, and osteoarthritis was admitted to the emergency department (ED) following a cardiac arrest. She had no documented pre-existing electrocardiographic abnormalities and no family history of sudden cardiac death or syncope. Over the past month, she had significant functional limitations due to a hip fracture, leading to reduced mobility. She had also been prescribed ciprofloxacin three days earlier for a urinary tract infection (UTI).

Earlier on the day of admission, while at home, she experienced sudden-onset diaphoresis and generalized weakness followed by loss of consciousness within minutes. Emergency medical services were called, and upon arrival, she was found unresponsive, pulseless, and in VF. Cardiopulmonary resuscitation (CPR) was initiated on-site, and she was immediately transferred to the ED.

Upon arrival at the ED, the patient remained in VF, and advanced cardiac life support protocols (ACLS) were initiated with concurrent intubation. Defibrillation was performed at 250 joules, but the patient remained in refractory VF, requiring repeated defibrillations after each rhythm check. She received four doses of epinephrine and 450 mg of amiodarone during CPR. After approximately 15 minutes and three more defibrillation shocks, return of spontaneous circulation (ROSC) was achieved, with a stable perfusing rhythm restored and no further recurrence of VF.

**Figure 1:**
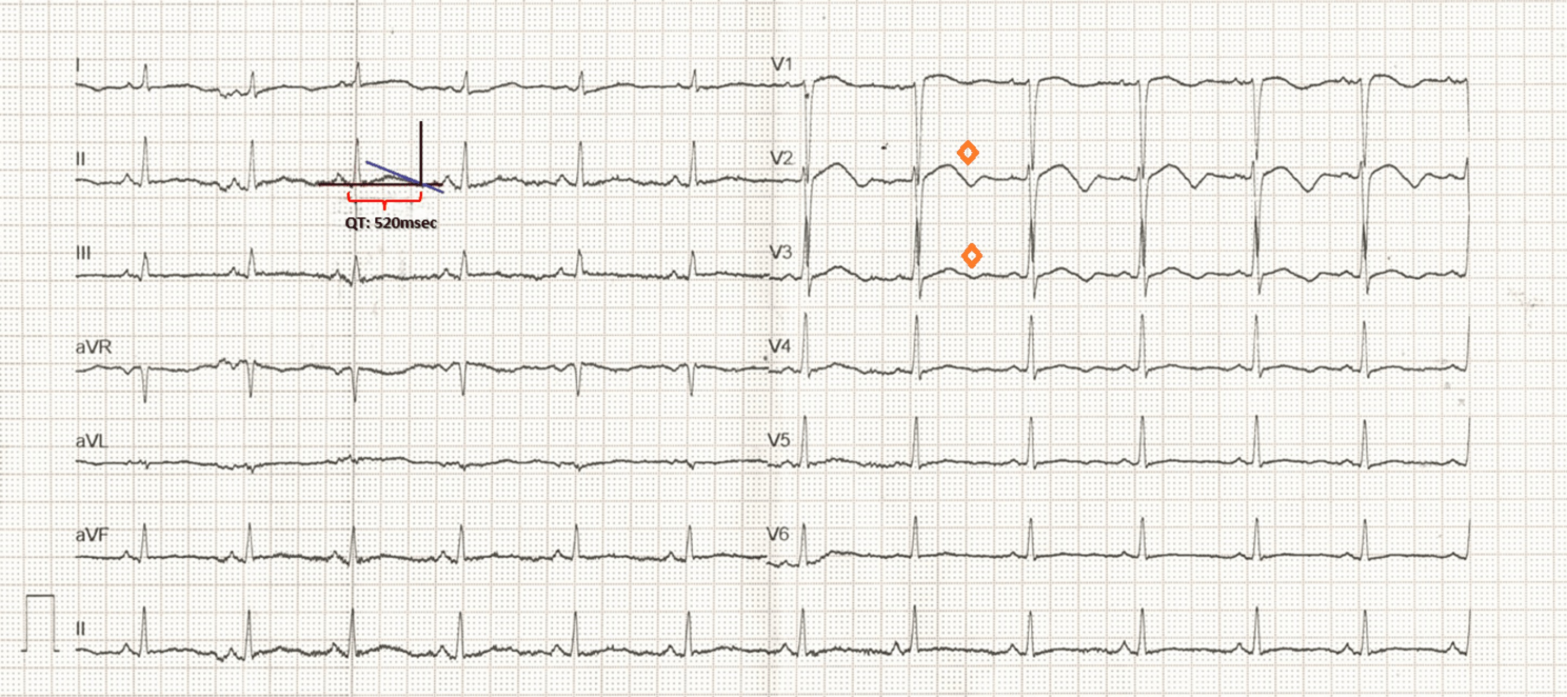
Post-ROSC ECG demonstrating sinus rhythm with a biphasic T wave in precordial lead V2, borderline in V3 (orange diamond), and significant QT prolongation (QTc: 581 milliseconds) using Bazett’s formula ROSC: return of spontaneous circulation; QTc: corrected QT interval

At the time of ROSC, the patient’s vital signs were as follows: blood pressure of 85/55 millimeters of mercury (mmHg), a heart rate of 80 beats per minute (bpm), and oxygen saturation of 96%, with a fraction of inspired oxygen set at 80%. Arterial blood gas analysis revealed lactic acidosis and mild hypokalemia. A post-ROSC ECG showed a biphasic T wave in precordial lead V2, borderline in V3, and significant QT prolongation (QTc: 581 msec) (Figure 1). Given the biphasic T waves in V2-V3, which met borderline criteria for Wellens' syndrome, occlusion of the left anterior descending artery (LAD) was initially considered a differential diagnosis. No pre-arrest ECG was available for comparison, making definitive exclusion of ischemic changes challenging at this stage. However, bedside echocardiography revealed preserved biventricular systolic function, no regional wall motion abnormalities, and no pericardial effusion. The absence of significant troponin elevation and typical angina symptoms further reduced the likelihood of acute coronary obstruction at that time.

Initial laboratory evaluation revealed mildly elevated inflammation markers (C-reactive protein (CRP): 3 milligrams per deciliter (mg/dl); upper normal limit: 0.5 mg/dl), high-sensitive troponin-I (hs-cTnI 250 picograms per milliliter (pg/ml), upper normal limit 38.7 pg/ml), mild hypokalemia (Potassium 3.2 milliequivalents per liter (mEq/L)), and evidence of UTI on urinalysis. No other significant findings were identified. The mild elevation in hs-cTnI was considered non-specific, as there was a borderline ischemic ECG pattern, and no regional wall motion abnormalities were observed. As part of the investigation of the aborted sudden cardiac death, brain, abdomen, and chest computed tomography (CT) was performed. These studies were negative for pulmonary embolism, acute aortic dissection, and other pathological findings involving the brain, lung parenchyma, and abdominal organs. Toxicology analysis as well as blood and urine cultures were obtained.

The patient was started on intravenous magnesium sulfate to reduce the risk of TdP, along with norepinephrine for hemodynamic support. Potassium repletion and intravenous crystalloids were administered to correct hypokalemia and restore intravascular volume. Intravenous broad-spectrum antibiotics were initiated for the UTI, while aspirin, clopidogrel, and subcutaneous fondaparinux were started for the probable acute coronary syndrome. Following the discovery of QT prolongation, ciprofloxacin, escitalopram, and amisulpride were discontinued.

Due to hemodynamic instability, the need for transfer to a hospital with a hemodynamic laboratory, and the absence of segmental wall motion abnormalities or significant troponin elevation, coronary angiography was initially deferred. The patient was admitted to the intensive care unit (ICU) for stabilization.

Within 24 hours, the patient was weaned off vasopressors and subsequently extubated. The patient remained hemodynamically and electrically stable, with no significant electrocardiographic changes for another 24 hours. Laboratory testing showed a downward trend of myocardial injury and inflammation markers. Based on the marked QT prolongation and the presence of multiple precipitating factors, including QT-prolonging medications (escitalopram, amisulpride, ciprofloxacin, and amiodarone administered during resuscitation), hypokalemia, and acute ischemia, acquired LQTS was considered the most likely diagnosis. Propranolol was initiated at a dose of 60 milligrams per day (mg/d).

After 48 hours of ICU care, the patient was transferred to the cardiology department for further evaluation and management.

**Figure 2:**
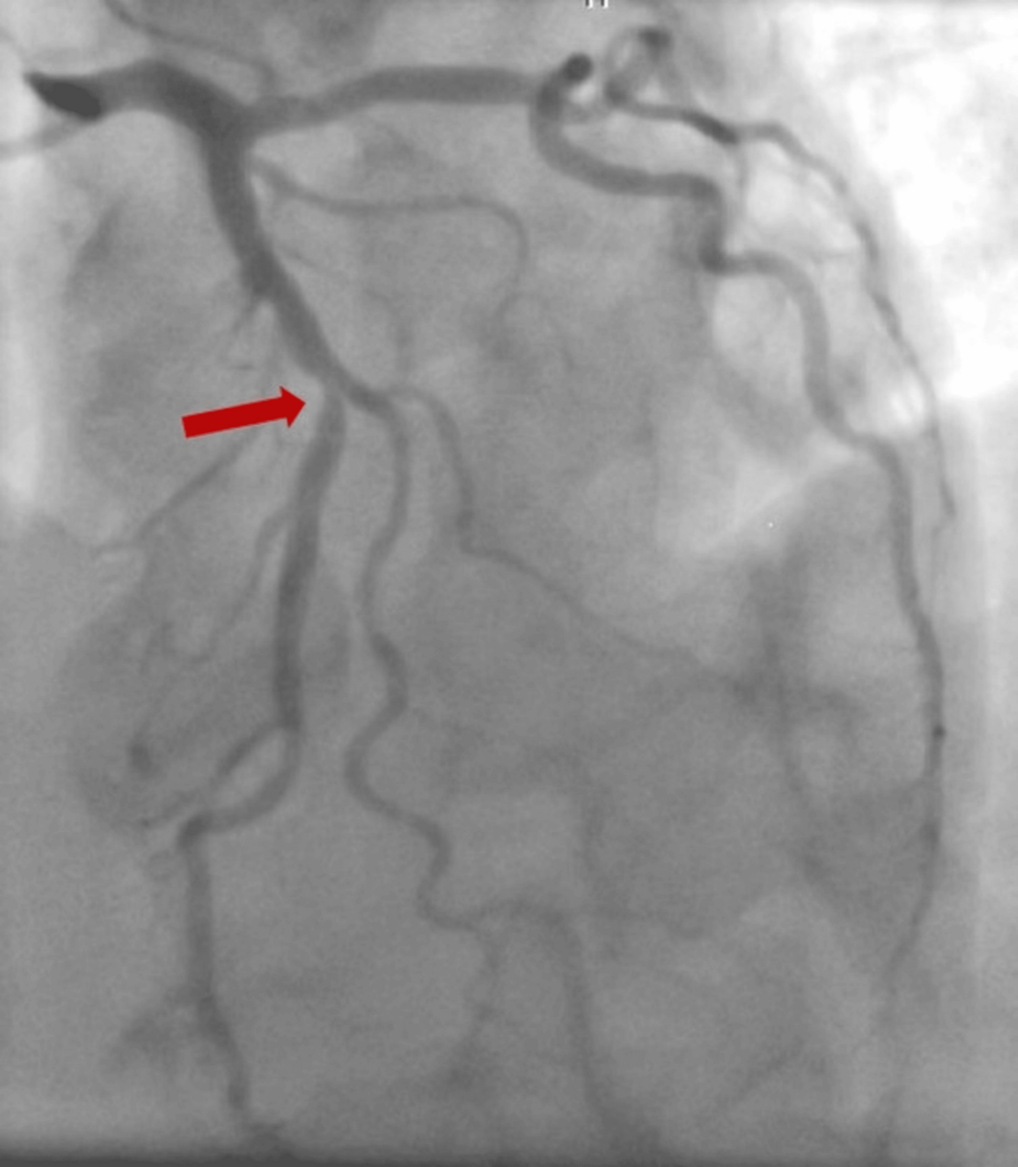
Coronary angiography showing a borderline stenosis in the middle segment of the left anterior descending artery (red arrow) with a fractional flow reserve (FFR) of 0.82, which did not warrant intervention, supporting the conclusion that ischemia was unlikely the primary cause of the arrest

Blood cultures were negative, while urine cultures identified *Escherichia coli *as sensitive to multiple antibiotics. Consequently, the antibiotic regimen was downgraded to oral cefuroxime. A complete transthoracic echocardiographic study revealed no significant findings, and on the third day of hospitalization, coronary angiography was performed, revealing borderline single-vessel disease (60%-70% mid-LAD stenosis; fractional flow reserve: 0.82). Given the absence of high-risk findings, conservative management was recommended, and clopidogrel monotherapy was continued with instructions for regular follow-up (Figure 2).

**Figure 3:**
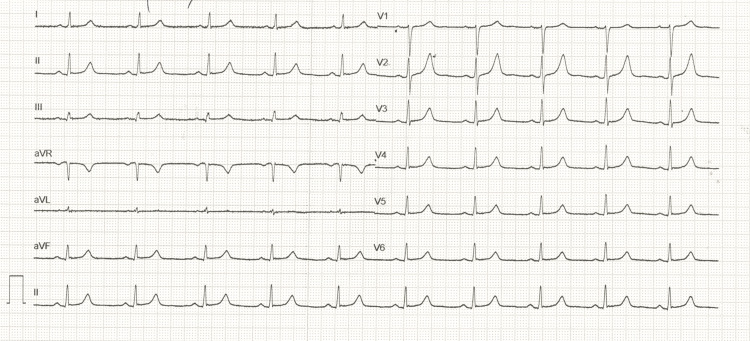
ECG with normal QT interval after discontinuation of QT-prolonging medications and correction of electrolyte imbalances (QTc: 440 milliseconds) QTc: corrected QT interval

A 24-hour Holter monitor showed no significant conduction abnormalities or complex ventricular arrhythmias. Continuous rhythm monitoring over the following days did not record any ventricular arrhythmias, while serial ECGs demonstrated gradual QT interval shortening after discontinuation of the offending drugs and correction of electrolyte disturbances (Figure 3). Neurology and psychiatry consultations were obtained, leading to the replacement of amisulpride and escitalopram with hydroxyzine, chosen for its sedative effects and minimal cardiac impact.

Screening of first-degree relatives was conducted, including detailed history-taking and ECGs, with no evidence of congenital LQTS identified. Based on the latest European Society of Cardiology (ESC) guidelines, an electrophysiological study was not indicated, and implantable cardioverter-defibrillator (ICD) placement was deemed unnecessary due to the presence of multiple reversible causes [8].

The patient remained hemodynamically stable throughout her hospitalization and was discharged with instructions to avoid QT-prolonging medication. She was advised to continue propranolol and consider genetic testing if feasible.

One year after the initial event, the patient remains asymptomatic, with no recurrence of arrhythmias on repeated Holter rhythm recordings. Genetic testing has not been performed yet, which remains a limitation in fully excluding an underlying predisposition to LQTS.

## Discussion

This case report highlights the synergistic effect of hypokalemia and multiple QT-prolonging medications (ciprofloxacin, amisulpride, escitalopram) in triggering acquired LQTS and subsequent cardiac arrest. The occurrence of drug-induced LQTS is inherently unpredictable, yet most affected patients have at least one identifiable risk factor in addition to drug exposure [5]. In this case, multiple risk factors for acquired LQTS were present, all contributing to the development of this condition. Female sex is a well-established risk factor for TdP, with women experiencing a two- to three-fold increased incidence compared to men. This disparity is attributed to hormonal influences on cardiac repolarization, particularly testosterone's role in enhancing the rapid component of the delayed rectifier potassium current (IKr). Testosterone shortens the QT interval in males, offering a protective effect against TdP, whereas females do not experience this benefit post-puberty, leaving them more susceptible to QT prolongation and arrhythmia [5,9].

In this patient, several medications contributed to QT interval prolongation. Escitalopram, a selective serotonin reuptake inhibitor (SSRI), can prolong the QT interval by blocking Kv 11.1 channels, impairing IKr current, and disrupting the trafficking of channel proteins to the cell membrane, ultimately delaying ventricular repolarization [10]. Ciprofloxacin, a fluoroquinolone antibiotic, also blocks the IKr current, delaying cardiac repolarization. Although ciprofloxacin carries a lower risk of QT prolongation compared to other fluoroquinolones, a few cases of TdP have been reported, particularly in patients with other risk factors [9,11]. Notably, one case involved a 76-year-old male patient with acute renal failure, hypocalcemia, and QT interval prolongation who received ciprofloxacin treatment and developed TdP triggered by hemodialysis [12]. Amisulpride, a selective dopamine D2 and D3 receptor antagonist, is generally safe at therapeutic doses but has been implicated in QT prolongation and TdP, especially at higher doses or in individuals with increased drug sensitivity. There is also a rare report of TdP occurring at low doses (100 mg/day) in a patient with a low body mass index, suggesting individual variability in drug sensitivity [13].

Hypokalemia is also a common and significant risk factor for drug-induced LQTS. Low extracellular potassium contributes to QT prolongation by impairing the outward potassium current during repolarization, which delays the return of the cell membrane to its resting state. This, in turn, amplifies the drug-induced IKr blockade. In this patient, the cumulative IKr blockade from multiple QT-prolonging agents, compounded by hypokalemia (K+ 3.2 mEq/L), significantly increased arrhythmogenic risk. Target potassium correction (>4.0 mEq/L) and magnesium administration were essential in reducing arrhythmic risk [5].

Ischemia consideration and coronary artery disease evaluation

Biphasic T waves in V2-V3 initially raised concerns for Wellens' syndrome and LAD occlusion. However, the absence of wall motion abnormalities, mild troponin elevation, and lack of preceding anginal symptoms made acute coronary occlusion less likely. Given the marked QT prolongation (QTc 581 ms) and multiple QT-prolonging factors, the clinical focus shifted toward stabilization and QT management. Coronary angiography was delayed due to hemodynamic instability and the need for transfer to another hospital due to the unavailability of angiography at our facility. The finding of borderline mid-LAD stenosis (60%-70%) with an FFR of 0.82 supported the decision for conservative management rather than urgent reperfusion therapy. However, immediate coronary angiography could have been a reasonable alternative, as ischemia remained a possible trigger.

Management of TdP and QT prolongation

The acute management of TdP depends on its clinical presentation. In hemodynamically unstable or sustained TdP, immediate electrical cardioversion is the treatment of choice. More commonly, TdP manifests as recurrent, self-terminating episodes. In these cases, the first-line treatment is intravenous administration of magnesium sulfate (2 grams over 1-2 minutes) with a repeat dose if necessary. Magnesium sulfate is effective in suppressing TdP without reducing the QT interval. The mechanism of action may be related to the suppression of late calcium influx via L-type calcium current and reduction in the amplitude of EADs. If magnesium sulfate fails, increasing the heart rate is the next step, typically achieved via transvenous pacing. Isoproterenol can be used temporarily to elevate the heart rate while awaiting pacing electrode insertion. Increasing the heart rate is associated with the shortening of the QT interval and suppression of TdP. Concurrently, any QT-prolonging should be discontinued, and acid-base and electrolytes should be corrected as necessary [3].

Long-term management involves educating patients about avoiding QT-prolonging drugs and providing a comprehensive list of such medications. In most cases, removing the causative drug and correcting other contributing factors normalizes the QTc interval. If the QTc interval remains prolonged, genetic testing for LQTS-associated mutations should be considered, particularly for symptomatic first-degree relatives of the patient [3].

In our case, the patient responded to magnesium and potassium repletion, avoiding the need for pacing or isoproterenol. Propranolol was continued, and patient education on QT-prolonging drugs was emphasized, including a medication review and a comprehensive list of high-risk drugs. Family screening was offered via ECG evaluation of first-degree relatives, with no abnormalities detected.

Congenital versus acquired LQTS

The Schwartz scoring system is a validated tool for assessing congenital LQTS, integrating clinical presentation, ECG findings, and family history [7].

On the other hand, the acquired form of LQTS is diagnosed on the basis of a QTc that exceeds 500 msec or when the QTc increases by >60 to 70 msec in the presence of medication, known as drug-induced LQTS, or another associated clinical precipitant [7]. However, up to 25% of patients with apparent acquired LQTS carry pathogenic variants in congenital LQTS genes, which may remain silent until triggered by external factors [13]. Genetic screening, particularly for mutations in KCNQ1, KCNH2, and SCN5A (responsible for LQTS1, LQTS2, and LQTS3, respectively), identifies mutations in 75% of cases, with these three genes accounting for 90% of positive findings [14].

In this case, other potentially reversible causes of acquired LQTS - such as myocardial ischemia, stroke, hypothyroidism, etc. - were effectively ruled out. Genetic testing was recommended to explore the possibility of congenital LQTS, but financial constraints have delayed testing, a common barrier in clinical practice. Alternative patient assistance programs or research initiatives could be explored to facilitate access. The lack of genetic testing remains a limitation in definitively ruling out an inherited predisposition. A positive genetic result could alter long-term management, including stronger consideration for ICD placement.

The patient was discharged with instructions to avoid QT-prolonging medications and to continue propranolol therapy. Ongoing follow-up and careful medication review remain crucial in preventing recurrence. To date, she remains asymptomatic, with no recurrence of arrhythmias.

## Conclusions

This case highlights that even in patients without a history of heart disease, attention should be paid to QTc prolongation, particularly in patients with pre-existing risk factors such as electrolyte imbalances, female sex, and polypharmacy. Early identification and mitigation of risk factors - including careful medication review, pharmacist consultation, and patient education - are critical in preventing life-threatening arrhythmias such as TdP and cardiac arrest. Patients should be educated on recognizing symptoms of arrhythmias and provided with accessible resources on QT-prolonging medications. Systematic strategies, such as regular ECG monitoring in high-risk individuals and standardized clinical protocols for managing QT-prolonging agents, should be implemented. Genetic testing may provide valuable insights into predisposition and guide long-term management, particularly in recurrent or high-risk cases.
